# Transcutaneous electrical acupoint stimulation versus sham stimulation and dexamethasone versus amisulpride for preventing postoperative nausea and vomiting after laparoscopic gynecologic surgery: protocol for a randomized 2 × 2 factorial trial

**DOI:** 10.3389/fphar.2026.1934841

**Published:** 2026-10-29

**Authors:** Yu Li, Jun Zhang, Yu Xu, Qin Liu, Lijuan Yang, Fuxiao Fang, Youjia Yu, Zhengjie Chen

**Affiliations:** 1 Department of Anesthesiology, Wenzhou Central Hospital, Wenzhou, Zhejiang, China; 2 Department of Anesthesiology, Deqing People’s Hospital, Huzhou, Zhejiang, China; 3 Department of Anesthesiology, Suzhou Xiangcheng People’s Hospital, Suzhou, China; 4 Operating Room, Deqing People’s Hospital, Huzhou, Zhejiang, China; 5 Department of Anesthesiology, Sir Run Run Shaw Hospital, Zhejiang University School of Medicine, Hangzhou, China

**Keywords:** acupuncture point, amisulpride, dexamethasone, postoperative nausea and vomiting, transcutaneous electrical stimulation

## Abstract

**Background:**

Postoperative nausea and vomiting (PONV) remains a common complication after gynecologic laparoscopic surgery. Although multimodal prophylaxis is recommended, clinical practice still relies largely on pharmacologic interventions. Transcutaneous electrical acupoint stimulation (TEAS) at the P6 acupoint may have potential benefit for PONV prevention. However, its effectiveness when incorporated into pharmacologic prophylaxis, and whether its effect varies according to the antiemetic used, remain unclear. This trial aims to evaluate the effect of P6-TEAS on PONV prevention in patients receiving dexamethasone or amisulpride.

**Methods:**

This multicenter, prospective, randomized, sham-controlled, 2 × 2 factorial trial will enroll 212 women undergoing gynecologic laparoscopic surgery under general anesthesia. Participants will be randomly assigned in a 1:1:1:1 ratio to receive one of four treatment combinations: TEAS plus dexamethasone, TEAS plus amisulpride, sham stimulation plus dexamethasone, or sham stimulation plus amisulpride. TEAS or sham stimulation will be delivered at the P6 acupoint in two 1-h sessions, before surgery and at 24 h after surgery. The primary outcome will be the incidence of PONV within 48 h after surgery. Secondary outcomes will include PONV-related symptoms and severity, use of rescue antiemetics, opioid consumption, pain scores, recovery milestones, patient satisfaction, QoR-15 scores, and length of hospital stay. Safety outcomes will include adverse events related to TEAS or study drugs and perioperative hemodynamic events requiring treatment.

**Discussion:**

This trial will evaluate P6-TEAS versus sham stimulation as the primary comparison and provide exploratory estimates for the dexamethasone-versus-amisulpride comparison and the TEAS-by-drug interaction. The findings may inform integrated prevention strategies for PONV after gynecologic laparoscopic surgery.

**Clinical Trial Registration:**

https://clinicaltrials.gov/study/NCT07631910, identifier NCT07631910.

## Introduction

Postoperative nausea and vomiting (PONV) is a common and clinically relevant complication after anesthesia and surgery (Rusch, Eberhart, Wallenborn and Kranke, 2010). Women undergoing gynecologic laparoscopic surgery are particularly susceptible to PONV because several established risk factors, including female sex, nonsmoking status, laparoscopic surgery, and perioperative opioid exposure, frequently coexist in this population (Apfel et al., 2012). In China, where smoking prevalence among women is low, a large proportion of patients undergoing gynecologic laparoscopic surgery may have at least moderate baseline risk of PONV (Tobacco Control OfficeChinese Center for Disease Control and Prevention, 2019). Although PONV is rarely life-threatening, it can delay oral intake and mobilization, impair patient comfort and postoperative recovery, prolong hospital stay, and increase healthcare utilization (Carroll, Miederhoff, Cox and Hirsch, 1994; Macario, Weinger, Carney and Kim, 1999).

To reduce the burden of PONV, current consensus guidelines recommend risk-stratified, multimodal prophylaxis, particularly for patients at moderate to high risk, using antiemetic interventions with different mechanisms of action (Gan et al., 2020). Recent multicenter evidence in patients undergoing gynecologic surgery further supports this multimodal approach, showing improved PONV control when fosaprepitant was added to palonosetron and dexamethasone (Zhang et al., 2024). Dexamethasone is an established first-line antiemetic and is widely used as part of combination prophylaxis (De Oliveira, Castro-Alves, Ahmad, Kendall and McCarthy, 2013; Gan et al., 2020). Dopamine receptor antagonists are another important class of antiemetic agents because they target dopaminergic signaling in the chemoreceptor trigger zone. However, the clinical use of older agents, such as droperidol and haloperidol, has been limited by concerns regarding sedation, extrapyramidal symptoms, and QT prolongation (Elias et al., 2023; Gan, 2007). Amisulpride, a selective dopamine D2/D3 receptor antagonist, has demonstrated efficacy for PONV prevention at low intravenous doses and offers a mechanistically distinct pharmacologic option for multimodal prophylaxis (Kranke et al., 2018).

Concerns about the adverse effects and incomplete efficacy of pharmacologic antiemetic prophylaxis have increased interest in nonpharmacologic strategies for PONV prevention. Current PONV guidelines endorse acupoint stimulation as one prophylactic option (Gan et al., 2020). However, traditional Neiguan (P6) stimulation techniques, such as needle acupuncture and acupoint injection, are invasive and may be associated with bleeding, infection, and limited perioperative acceptability. Transcutaneous electrical acupoint stimulation (TEAS) at P6 provides a noninvasive alternative. Accumulating perioperative evidence suggests that P6-TEAS may have antiemetic effects (Lee et al., 2025; Stoicea et al., 2015). In patients undergoing gynecologic laparoscopic surgery, P6-TEAS combined with dexamethasone has been reported to reduce 24-h PONV compared with dexamethasone alone (Yang et al., 2015). However, the preventive effect of P6-TEAS has not been consistent across clinical settings (Gan, Jiao, Zenn and Georgiade, 2004; Jin et al., 2025; Woodward et al., 2024), and whether its role in PONV prophylaxis differs across pharmacologic regimens in patients undergoing gynecologic laparoscopic surgery remains unclear.

Women undergoing gynecologic laparoscopic surgery remain at substantial risk of PONV despite pharmacological prophylaxis, supporting the need for additional preventive strategies within a multimodal approach. Although P6-TEAS is a promising nonpharmacological intervention, its additional benefit when used with active antiemetic prophylaxis remains uncertain. We therefore designed a multicenter, randomized, participant- and outcome assessor-blinded, sham-controlled, 2 × 2 factorial trial.

## Methods

### Study design and patients

This is a multicenter, prospective, randomized, sham-controlled, 2 × 2 factorial clinical trial. The primary estimand is the intention-to-treat effect of assignment to P6-TEAS versus sham stimulation on cumulative 48-h PONV. The effect of assignment to dexamethasone versus amisulpride is prespecified as exploratory. The two prespecified comparisons are: (1) P6-TEAS versus sham stimulation, with participants pooled across drug allocation; (2) dexamethasone versus amisulpride, with participants pooled across stimulation allocation. The trial will be conducted at Sir Run Run Shaw Hospital, Zhejiang University School of Medicine, Wenzhou Central Hospital, and Deqing County People’s Hospital. Participant recruitment is scheduled from 1 July 2026, to 31 October 2026.

A total of 212 eligible participants will be randomly assigned in a 1:1:1:1 ratio to one of four treatment combinations: TEAS plus dexamethasone, TEAS plus amisulpride, sham stimulation plus dexamethasone, or sham stimulation plus amisulpride. The study flow diagram is shown in Figure 1, and the schedule of enrollment, interventions, and outcome assessments is presented in Table 1.

**FIGURE 1:**
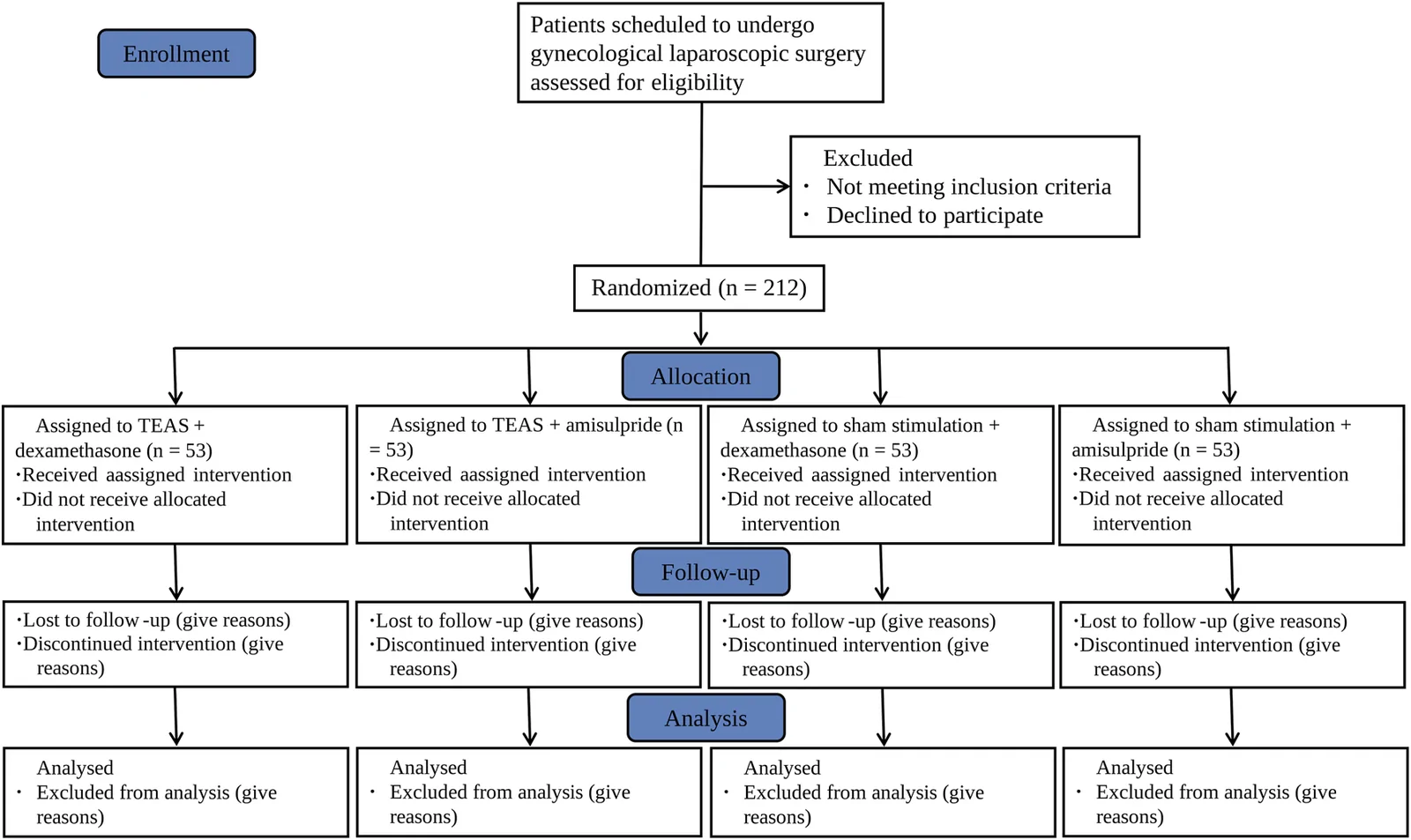
Planned participant-flow diagram.

**TABLE 1:** Schedule of patient enrolment, study interventions and outcome assessment.

Time point	Study period						
	Enrolment	Allocation	Post-allocation				Close-out
	Pre-op visit	Pre-anesthesia	Intraoperatively	PACU	24h post-op	48h post-op	Discharged
Patient enrolment							
Eligibility criteria	×	​	​	​	​	​	​
Written informed consent	×	​	​	​	​	​	​
Demographic data	×	​	​	​	​	​	​
Baseline characteristics	×	​	​	​	​	​	​
Randomization	​	×	​	​	​	​	​
Allocation	​	×	​	​	​	​	​
Study interventions							
Dexamethasone	​	​	×	​	​	​	​
Amisulpride	​	​	×	​	​	​	​
TEAS	​	×	​	​	×	​	​
Sham stimulation	​	×	​	​	×	​	​
Outcome assessment							
PONV	​	​	​	×	×	×	​
Severity of PONV	​	​	​	×	×	×	​
Nausea	​	​	​	×	×	×	​
Vomiting	​	​	​	×	×	×	​
Retching	​	​	​	×	×	×	​
Time to first flatus	​	​	​	​	×	×	​
Time to first oral intake	​	​	​	​	×	×	​
Time to first ambulation	​	​	​	​	×	×	​
NRS pain scores at rest	​	​	​	​	×	×	​
NRS pain score during movement	​	​	​	​	×	×	​
Need for rescue antiemetics	​	​	​	​	×	×	​
NRS satisfaction score	​	​	​	​	​	×	​
Opioid consumption	​	​	​	​	​	×	​
Need for rescue analgesia	​	​	​	​	×	×	​
QoR-15 scores	​	​	​	​	×	​	​
Length of postoperative stay	​	​	​	​	​	​	×
Safety outcomes							
Local skin allergy (redness, swelling, or numbness)	​	×	​	​	×	×	​
Localized skin burning or pain	​	×	​	​	×	×	​
Itching at the electrode site	​	×	​	​	×	×	​
Headache	​	​	​	​	×	×	​
Dizziness	​	​	​	​	×	×	​
Interventions for hemodynamic events	​	​	×	×	×	×	​
Hypersensitivity reactions	​	​	×	×	×	×	​
Extrapyramidal symptoms	​	​	×	×	×	​	​
Sedation	​	​	​	×	​	​	​
Postoperative hyperglycemia	​	​	​	​	×	​	​
Clinically relevant arrhythmias	​	​	×	×	×	×	​

PACU, Post-Anesthesia Care Unit; TEAS, transcutaneous electrical acupoint stimulation; PONV, postoperative nausea and vomiting; Pre-op, Preoperative; Post-op, Postoperative; NRS, numerical rating scale; QoR-15, scores, Quality of Recovery-15.

The current study protocol (version 1.0, dated 6 January 2026) and related documents were approved by the Ethics Committee of Sir Run Run Shaw Hospital, Zhejiang University School of Medicine (No. 20260278; 19 March 2026), the Ethics Committee of Wenzhou Central Hospital (No. 202607031618000408946; 3 July 2026), and the Ethics Committee of Deqing People’s Hospital (No. LL2026-DRY-X029; approval date: 23 July 2026). Written informed consent will be obtained from all participants before enrollment and before any trial-related procedures. Participants will be informed of the study purpose, procedures, potential risks and benefits, and their right to withdraw at any time without affecting their medical care. For participants with limited literacy, the approved study information and consent form will be read aloud and explained in clear, nontechnical language. Sufficient time will be provided for questions, and understanding will be confirmed before consent. If a participant cannot read the consent form, an impartial witness will attend the consent discussion and sign the form. The participant will provide a signature or legally acceptable mark and receive a copy of the completed form. The trial has been registered at ClinicalTrials.gov (NCT07631910).

### Inclusion criteria

- Aged 18–65 years;
- ASA physical status I-II;
- Scheduled to undergo gynecologic laparoscopic surgery under general anesthesia;
- Written informed consent provided.

### Exclusion criteria

- Severe cardiovascular, hepatic, renal, pulmonary, or other systemic diseases;
- Allergy to TEAS or skin disease at the stimulation site;
- Receipt of antiemetics within 24 h before surgery;
- Implanted cardiac pacemaker, cardioverter-defibrillator, or other electronic medical device;
- Pregnancy or lactation;
- Chemotherapy or radiotherapy within 4 weeks before surgery;
- QTcF (QT/RR^1/3^) >470 m on 12-lead electrocardiogram
- Vulnerable populations, including critically ill patients, patients with psychiatric disorders, cognitive impairment, or illiteracy.

### Primary outcome

The primary outcome is the incidence of PONV, defined as the proportion of participants experiencing at least one episode of nausea, retching, or vomiting from the end of surgery through 48 h postoperatively. PONV will be considered present if any nausea, retching, or vomiting occurs. Nausea will be defined as a subjective, unpleasant sensation associated with the urge to vomit; retching as labored, spasmodic respiratory or abdominal movements without the expulsion of gastric contents; and vomiting as the forceful expulsion of gastric contents. The end of surgery will be defined as time zero. Blinded outcome assessors will evaluate PONV during three prespecified, nonoverlapping intervals: from the end of surgery to PACU discharge, from PACU discharge to 24 h after surgery, and from 24 to 48 h after surgery. During the PACU stay, nausea, retching, and vomiting will be assessed through participant self-report and contemporaneous direct observation. At the 24- and 48-h assessments, a standardized interview will cover the entire interval since the preceding assessment, and the medical and nursing records will be reviewed for additional documented nausea, retching, or vomiting. The 0–24-h outcome will be derived from the first two intervals, whereas PONV during the 0–48-h period will be considered present if at least one qualifying event occurs during any of the three intervals, irrespective of rescue antiemetic treatment.

### Secondary outcomes

Secondary outcomes will include the severity of nausea, severity of PONV, time to first flatus, time to first oral intake, time to first ambulation, use of rescue antiemetics, additional analgesic requirements, cumulative postoperative opioid consumption, pain intensity at rest and during movement, quality of recovery (QoR-15), patient satisfaction, and length of hospital stay.

At each of the three prespecified assessment time points, interval-specific PONV incidence will be assessed and reported separately as a secondary outcome, while the simplified PONV impact scale will be completed for the corresponding interval to assess clinically important PONV. PONV symptoms will additionally be classified using the following study-defined categories: 0, no nausea, retching, or vomiting; 1, nausea only; 2, retching or vomiting; and 3, refractory vomiting (Myles and Wengritzky, 2012). Among participants who experience nausea, symptom severity will be assessed using an 11-point numeric rating scale (NRS), ranging from 0 (no nausea) to 10 (the worst imaginable nausea). Each assessment will cover symptoms occurring throughout the corresponding interval.

Time to first flatus, first oral intake, and first ambulation will be recorded within the first 24 h after surgery. Rescue antiemetic use, additional analgesic requirements, and cumulative postoperative opioid consumption will be recorded during the first 48 postoperative hours. Pain intensity at rest and during movement will be assessed using an 11-point NRS at 24 and 48 h postoperatively. Quality of recovery will be assessed using the QoR-15 at 24 h after surgery. Patient satisfaction will be assessed at 48 h using an 11-point NRS.

### Safety outcomes

Safety outcomes will include local skin reactions at the stimulation site, localized burning or pain, headache, dizziness, perioperative hemodynamic events requiring treatment, clinically relevant arrhythmias, extrapyramidal symptoms, sedation, severe hypersensitivity reactions, and postoperative hyperglycemia.

Hemodynamic interventions will include the use of ephedrine, metaraminol, atropine, urapidil, or esmolol. Safety outcomes will be assessed and documented throughout the hospital stay after the study intervention. Clinically relevant arrhythmias will be defined as new-onset sustained supraventricular or ventricular arrhythmias, torsades de pointes, or bradyarrhythmias or tachyarrhythmias associated with symptoms, hemodynamic instability, or a requirement for treatment. A confirmed QTcF interval of ≥500 m or an increase of ≥60 m from baseline will also be recorded as a prespecified cardiac safety event. Extrapyramidal symptoms will include new-onset akathisia, acute dystonia, parkinsonism, or dyskinesia. Clinically relevant sedation will be defined as unexpected or prolonged sedation that delays discharge from the PACU or requires pharmacologic reversal, airway support, or ventilatory assistance. Severe hypersensitivity reactions will include anaphylaxis, angioedema, bronchospasm, or generalized allergic reactions requiring epinephrine, airway management, vasopressor support, or prolongation of hospitalization.

### Randomization and blinding

An independent biostatistician who is not involved in participant recruitment or outcome assessment will generate the randomization sequence using the Sealed Envelope online randomization system (https://www.sealedenvelope.com/simple-randomiser/v1/lists), with randomly permuted blocks of sizes four and 8 and stratification by study center. Allocation will be concealed using center-specific, sequentially numbered, opaque, sealed envelopes, which will be opened by a designated unblinded study coordinator only after eligibility has been confirmed and the participant has arrived in the operating room. Emergency unblinding will be permitted only when knowledge of treatment allocation is essential for clinical management and will be documented.

Participants will be blinded to both the stimulation and pharmacological allocations throughout the study. Surgeons, anesthesiologists responsible for perioperative management and study medication administration, postoperative outcome assessors, and statisticians will also remain blinded to both allocations. The unblinded medication-preparation coordinator will prepare dexamethasone or amisulpride according to the randomization schedule and will therefore be aware of the pharmacological allocation, but will remain blinded to the stimulation allocation. The administering anesthesiologist will receive an identically appearing 5-mL syringe and will remain blinded to its contents. The TEAS operator will be aware of the stimulation allocation because the stimulation procedure differs between active TEAS and sham stimulation, but will not be informed of the pharmacological allocation. For participants in the active P6-TEAS group, the TEAS operator will adjust stimulation intensity according to the participant’s reported sensation and tolerability. No intensity adjustment will be performed in the sham group. Postoperative outcome assessors will not participate in device setup or intensity adjustment. Intervention records containing allocation-revealing information will be maintained separately from records available for blinded outcome assessment. The TEAS operator and medication-preparation coordinator will have no role in postoperative follow-up, outcome assessment, data management, or statistical analysis and will be instructed not to disclose allocation information.

Both groups will undergo the same device-placement procedures and receive standardized communication, comparable staff contact, and safety checks. The sham device will not deliver electrical stimulation at any time during the intervention, while its light-emitting diode (LED) indicators will mimic the operational cues of the active device. Before study implementation, participant-visible differences in indicator lights, displays, audible signals, or other operational cues between active and sham conditions will be checked. Where feasible and consistent with safe device operation, verified masking measures will be applied identically in both groups to minimize participant-visible allocation cues.

Before each session, participants will receive the same standardized and neutral explanation: “The strength and perception of sensation may vary among individuals during the session. A weaker or less noticeable sensation does not establish which study intervention you are receiving. Please tell the study staff if you experience pain, skin discomfort, or any other discomfort.” This explanation will not imply that electrical output necessarily continues throughout the session. Participants will not be informed whether they are receiving active or sham stimulation.

At 48 h postoperatively, after the scheduled PONV assessment and before allocation disclosure, an assessor blinded to stimulation assignment will ask participants whether they believe they received active stimulation or sham stimulation, or whether they do not know. Response distributions and the Bang blinding index with its 95% confidence interval will be reported separately for the active and sham stimulation groups.

### Study interventions

Participants will receive active P6-TEAS or sham stimulation according to their randomized stimulation assignment, together with their assigned antiemetic. The same stimulation procedures will be used across both antiemetic assignments. Trained intervention personnel will administer all sessions according to a standardized operating procedure across study centers. A wearable TEAS wristband (EmeTerm, model YF-ZTY-E1; WAT Medical Enterprise) will be applied to the P6 acupoint on the volar forearm of the dominant upper extremity. The P6 acupoint is located approximately 3–5 cm proximal to the distal wrist crease, between the tendons of the flexor carpi radialis and palmaris longus. Before each session, the application site, skin, and device will be checked and prepared in accordance with the manufacturer’s instructions. The stimulating electrodes are integrated into the wristband, eliminating the need for external adhesive electrodes. Electrode contact and wristband fit will also be checked before each session. According to the manufacturer’s specifications, the device has five adjustable intensity levels, a stimulation frequency of 33 ± 3 Hz, and a specified output-voltage amplitude range of −42 to +42 V. Stimulation intensity will be indicated by five LED indicators: illumination of one LED will correspond to the lowest intensity level (level 1), whereas illumination of all five LEDs will correspond to the highest intensity level (level 5), with intermediate numbers of illuminated LEDs indicating the corresponding intermediate intensity levels.

TEAS or sham stimulation will be administered by trained intervention personnel at two time points: before surgery and 24 h after surgery, with each session lasting 60 min. At the 24-h time point, the prespecified outcome assessment will be completed first, after which the second 60-min TEAS or sham-stimulation session will be initiated.

In the active P6-TEAS group, stimulation will begin at level 1 and will be increased stepwise according to the participant’s reported sensation and tolerability. The highest available level that produces a perceptible but comfortable sensation without pain will be selected and maintained throughout the remainder of the treatment session. Intensity will be reduced if discomfort occurs, and stimulation will be interrupted or discontinued if discomfort persists or becomes intolerable.

In the sham group, the device will be placed at the same anatomical site using the same placement procedure and session schedule as in the active group, but no electrical stimulation will be delivered at any time during the session. The sham device will reproduce the external appearance and operational cues of the active device, including the LED display, while electrical output remains disabled throughout the 60-min session.

For participants in the active P6-TEAS group, each session will include an initial intensity-adjustment phase. Actual session times, device placement, stimulation intensity in the active group, discomfort, interruptions, and reasons for premature discontinuation will be documented. Protocol-defined rescue antiemetic treatment will not be delayed or withheld because of a stimulation session. Participants who discontinue stimulation will continue outcome follow-up.

Participants will receive dexamethasone or amisulpride according to their randomized allocation. Study medications will be prepared in identical syringes by a designated unblinded study coordinator according to the randomization schedule, thereby maintaining blinding of participants and the administering anesthesiologist.

#### Anaesthetic care

All participants will fast for at least 8 h before surgery, and no premedication will be administered. Upon arrival in the operating room, standard monitoring will include electrocardiography, noninvasive blood pressure, pulse oximetry, capnography, and bispectral index (BIS). General anesthesia will be induced with intravenous propofol (2–3 mg/kg), sufentanil (0.3–0.5 μg/kg), and cisatracurium (0.15 mg/kg) to facilitate endotracheal intubation. Following intubation, patients will receive mechanical ventilation adjusted to maintain normocapnia.

Anesthesia will be maintained with sevoflurane-based balanced general anesthesia using oxygen and air, supplemented with intravenous propofol, remifentanil, and dexmedetomidine. Drug dosages will be titrated according to clinical requirements to maintain a BIS value between 40 and 60. Additional cisatracurium will be administered as required to maintain adequate neuromuscular blockade. All procedures will be performed under carbon dioxide pneumoperitoneum. Intravenous lactated Ringer’s solution and blood transfusion will be administered according to intraoperative clinical requirements. To minimize between-center variability in co-interventions, all three centers will follow the same anesthetic protocols; deviations from the prespecified anesthetic regimen will be documented.

At the time of induction of anesthesia, participants allocated to the dexamethasone groups will receive intravenous dexamethasone (5 mg), whereas those allocated to the amisulpride groups will receive intravenous amisulpride (5 mg). No additional prophylactic antiemetic agents will be administered intraoperatively. No routine postoperative prophylactic antiemetics will be given during the 48-h primary-outcome period; antiemetics administered during this period will be limited to protocol-defined rescue treatment. Intravenous flurbiprofen axetil (50 mg) will be administered at the end of surgery for postoperative analgesia.

Perioperative hemodynamic events requiring treatment, including clinically significant hypotension, hypertension, bradycardia, and tachycardia, will be managed according to standard institutional practice and recorded. Vasoactive medications may include ephedrine, metaraminol, atropine, urapidil, or esmolol, as clinically indicated. The attending anesthesiologist will document intraoperative anesthetic drug consumption, fluid administration, estimated blood loss, anesthesia duration, surgical duration, hemodynamic interventions, vasoactive medication use, and any conversion to open surgery.

After surgery, participants will be transferred to the post-anesthesia care unit (PACU). Residual neuromuscular blockade will be assessed according to routine clinical practice, and pharmacologic reversal will be administered when clinically indicated. Tracheal extubation will be performed after standard extubation criteria are fulfilled. Rescue analgesia will consist of intravenous oxycodone (5 mg) for an NRS pain score >4 at rest or during movement. Rescue antiemetic therapy will consist of intravenous ondansetron (4 mg) if the nausea NRS score is ≥ 7, vomiting occurs, or the participant requests treatment. First-line rescue treatment will consist of intravenous ondansetron 4 mg administered over 2–5 min, with treatment response reassessed after 30 min. Refractory vomiting will be defined as vomiting or retching that persists or recurs within 30 min after ondansetron and will be treated with promethazine 6.25 mg by deep intramuscular injection. If symptoms initially resolve but subsequently recur, ondansetron 4 mg may be repeated once after at least 6 h, with a maximum cumulative dose of 8 mg within 24 h. Ondansetron will be administered under continuous electrocardiographic monitoring during administration and for at least 1 h thereafter; confirmed QTcF ≥500 m or an increase ≥60 m from baseline will prompt withholding of further QT-prolonging medications and appropriate clinical evaluation. The same rescue protocol will be used at all centers. If symptoms persist after promethazine, additional antiemetic treatment may be administered at the attending anesthesiologist’s discretion when clinically necessary and will be documented as nonprotocol rescue treatment. PONV assessments will continue after rescue treatment. Each episode of nausea, vomiting, or retching will be recorded with onset time, type, and nausea severity (0-10 numerical rating scale), and rescue antiemetic use will be documented with the indication, agent, dose, route, and administration time. Symptoms recurring after complete resolution for at least 30 min will be recorded as a new episode. Length of hospital stay will be obtained from the electronic medical record system.

### Data collection and monitoring

Baseline characteristics will include, but will not be limited to, age, height, weight, body mass index, smoking status, history of postoperative nausea and vomiting or motion sickness, comorbidities, current medications, drug allergy history, planned surgical procedure, preoperative laboratory results, Apfel PONV risk score, and American Society of Anesthesiologists (ASA) physical status classification.

Blinded investigators will collect study data throughout the perioperative period and follow-up, including primary outcome data, secondary outcome data, safety outcomes, and other relevant perioperative variables. All data will be recorded in standardized case report forms (CRFs) and subsequently entered into a secure, password-protected electronic database. Data quality will be ensured through regular review for completeness, consistency, and accuracy.

Participant and data flow will be documented from screening through randomization, intervention delivery, 48-h follow-up, and analysis, with reasons for exclusion, withdrawal, protocol deviations, and missing outcome assessments recorded. Source data from participant interviews, PACU observation, anesthesia and medication records, intervention logs, and nursing records will be cross-checked against the case report forms for completeness and consistency. Any identified discrepancies will be verified against the relevant source records and resolved before database lock and final analysis.

According to the prespecified statistical analysis plan, all analyses will be performed by an independent statistician using a fully de-identified dataset. An independent Data and Safety Monitoring Committee (DSMC) will oversee trial conduct, monitor protocol adherence, review data quality, and periodically evaluate participant safety throughout the study.

#### Sample size calculation

The sample size calculation is based on the primary comparison of 48-h PONV incidence between participants receiving TEAS and sham stimulation, pooled across drug allocation. Previous randomized trials in women undergoing gynecologic laparoscopic surgery reported PONV incidences of approximately 50% in control or dexamethasone-only groups and 17.5%–28% in TEAS-based intervention groups (Jin et al., 2025; Yang et al., 2015). Assuming 48-h PONV rates of 50% with sham stimulation and 30% with P6-TEAS, a two-sided α of 0.05, and 80% power, a conventional two-proportion calculation requires approximately 93 evaluable participants in each stimulation group. With balanced allocation across the two antiemetic assignments, this corresponds to 46.5 participants per factorial treatment combination, which is rounded up to 47. Allowing for 10% of participants to have unavailable primary-outcome data, 47/0.90 = 52.2 participants are required per treatment combination, which is rounded up to 53. Thus, the final target sample size is 212 participants, with 53 participants in each of the four factorial treatment combinations. This sample size is intended to provide adequate power for the main-effect comparison of P6-TEAS versus sham stimulation, assuming no clinically important P6-TEAS-by-antiemetic interaction. The trial is not specifically powered for the dexamethasone-versus-amisulpride comparison or for the P6-TEAS-by-antiemetic interaction; these analyses will therefore be considered exploratory.

### Statistical analysis

A prespecified statistical analysis plan will be finalized before database lock and unblinding. Continuous variables will be assessed for normality using the Shapiro-Wilk test and visual inspection of distributional plots. Data will be summarized as mean (standard deviation), median (interquartile range), or number (percentage), as appropriate. Baseline characteristics, perioperative variables, and safety outcomes will be described by the four factorial treatment groups. Formal statistical testing of baseline characteristics will not be performed.

The primary analysis will focus on the effect of P6-TEAS, compared with sham stimulation, on the occurrence of PONV within 48 h after surgery, pooling participants across the two antiemetic assignments. A logistic regression model will include stimulation assignment, antiemetic assignment, and study center as fixed effects, without an interaction term. The effect of stimulation will be reported as an adjusted odds ratio (OR) with a 95% confidence interval (CI). Model-based predicted probabilities and the corresponding absolute risk difference with a 95% CI will also be reported as supplementary measures for clinical interpretability.

The primary efficacy analysis will follow the intention-to-treat principle and target all randomized participants according to their assigned treatment. Treatment discontinuation, nonadherence, crossover, or protocol deviations will not result in exclusion from the intention-to-treat analysis, and follow-up will continue whenever consent is maintained. Participants with missing primary-outcome data will remain part of the intention-to-treat estimand and will be handled as prespecified below. Major protocol deviations will be prespecified before database lock, and a per-protocol analysis excluding participants with major deviations will be performed as a sensitivity analysis. PONV assessments will continue after rescue treatment, and all qualifying events within 48 h will contribute to the primary outcome.

Given the factorial design, a separate logistic regression model will include a stimulation-by-antiemetic interaction term in addition to stimulation assignment, antiemetic assignment, and study center. The interaction will be assessed using a two-sided Wald test. The exponentiated interaction coefficient will be reported as the ratio of the OR for P6-TEAS versus sham stimulation in the dexamethasone group to the corresponding OR in the amisulpride group, with its 95% CI, together with the ORs and 95% CIs for the effect of stimulation within each antiemetic group. Because the sample size is not determined to detect an interaction, interpretation will emphasize the magnitude and precision of the effect estimates; a statistically nonsignificant interaction test will not be interpreted as evidence of no interaction.

The main effect of antiemetic assignment will be evaluated as a prespecified secondary analysis using the primary regression model. PONV outcomes will also be summarized descriptively for the four groups. Because the sample size is determined for the stimulation comparison rather than the antiemetic comparison, the trial may provide insufficient power to detect smaller differences between the antiemetics. No adjustment for multiple testing will be made for the antiemetic comparison or the interaction analysis; these results will therefore be interpreted as exploratory.

Secondary outcomes will be analyzed according to their scale and longitudinal structure. Unless otherwise specified, regression models will include stimulation assignment, antiemetic assignment, and study center as prespecified covariates, consistent with the primary analysis framework. Continuous outcomes will be analyzed using linear regression when model assumptions are reasonably satisfied and appropriate nonparametric methods otherwise. Binary outcomes will be analyzed using logistic regression, and ordinal outcomes, including PONV severity, will be analyzed using ordinal logistic regression where the proportional-odds assumption is appropriate. Repeated-measures outcomes, including pain scores and PONV severity assessed over time, will be analyzed using models accounting for within-participant correlation. Effect estimates will be reported with corresponding 95% confidence intervals where appropriate.

The extent, patterns, and documented reasons for missing primary-outcome data will be summarized by randomized group. If fewer than 5% of participants have missing primary-outcome data and the documented reasons support the assumption that outcome missingness is independent of PONV status conditional on the covariates in the primary analysis model, the primary analysis will include participants with observed primary outcomes without imputation. Otherwise, missing primary-outcome data will be handled using multiple imputation by chained equations under a missing-at-random assumption, generating 50 imputed datasets. The imputation model will include stimulation assignment, antiemetic assignment, their interaction, study center, relevant baseline characteristics, and available PONV data from each assessment interval. Regression estimates will be pooled using Rubin’s rules. When primary outcome data are missing, analyses using observed outcomes and multiple imputation will be compared to assess sensitivity to the handling of missing data. Sensitivity analyses allowing departures from the missing-at-random assumption will also be conducted using extreme-case scenarios. In one scenario, all missing outcomes in the P6-TEAS group will be classified as PONV and all missing outcomes in the sham-stimulation group as no PONV; the classifications will be reversed in the other scenario.

Blinding will be evaluated using the Bang blinding index with its 95% confidence interval. Safety analyses will include all participants who receive at least one study intervention, and adverse events will be summarized by treatment group, type, and severity. All tests will be two-sided, with P < 0.05 considered statistically significant for the main TEAS-versus-sham analysis. Statistical analyses will be performed using SPSS version 25.0 (IBM Corp., Armonk, NY, United States of America).

## Discussion

This multicenter, randomized, sham-controlled, participant-, assessor-, and statistician-blinded 2 × 2 factorial trial will enroll 212 women across three centers to evaluate P6-TEAS and pharmacologic prophylaxis with dexamethasone or amisulpride for the prevention of PONV after gynecologic laparoscopic surgery under general anesthesia. The primary outcome is the incidence of any PONV within the first 48 h after surgery. Secondary outcomes will assess nausea severity, PONV severity, rescue medication use, postoperative analgesic requirements, pain intensity, quality of recovery, patient satisfaction, recovery milestones, and length of hospital stay. The protocol follows the Standard Protocol Items: Recommendations for Interventional Trials (SPIRIT) guidelines (Chan et al., 2025).

Previous randomized trials have supported the potential role of TEAS in women undergoing gynecologic laparoscopic surgery. Yang et al. showed that P6 electrical acupoint stimulation combined with dexamethasone reduced 24-h PONV compared with dexamethasone alone, with an antiemetic effect similar to tropisetron plus dexamethasone (Yang et al., 2015). More recently, Jin et al. reported that TEAS, particularly multi-period stimulation, reduced PONV and showed additional signals of improved pain control and gastrointestinal recovery in this population (Jin et al., 2025). However, these studies mainly evaluated TEAS within a fixed pharmacologic background or focused on different TEAS timing regimens. Building on previous studies using different TEAS timing regimens, the present protocol uses a wearable P6-TEAS device with individually titrated intensity and two 60-min sessions administered before surgery and 24 h postoperatively. Individualized intensity adjustment is intended to maintain participant comfort, while the two-session schedule extends stimulation into the early postoperative period. Combining P6-TEAS with dexamethasone or amisulpride is consistent with the principle of multimodal PONV prophylaxis and is supported by guideline recommendations and previous evidence (Gan et al., 2020; Yang et al., 2015).

The inclusion of dexamethasone and amisulpride is clinically relevant because these agents represent different antiemetic mechanisms. Dexamethasone is widely used as part of multimodal PONV prophylaxis and has been supported by extensive clinical evidence (Apfel et al., 2004; Henzi, Walder and Tramer, 2000). Amisulpride, a selective dopamine D2/D3 receptor antagonist, targets dopaminergic signaling and provides a mechanistically distinct pharmacologic option (Fox, Albayaty, Walker, Xue and Darpo, 2021; Gan et al., 2017; Taubel et al., 2017; Zhang et al., 2020). Amisulpride was selected to provide a mechanistically distinct pharmacological context rather than because of presumed superiority over other antiemetics. Although both dexamethasone 5 mg and amisulpride 5 mg are clinically relevant prophylactic regimens within guideline-supported dose ranges, they are not assumed to be therapeutically equivalent or equipotent. Evaluating TEAS across these two pharmacologic contexts may provide more informative evidence than repeating another trial of TEAS within a single dexamethasone-based regimen. The sample size was based on the primary TEAS-versus-sham comparison. Accordingly, the dexamethasone-versus-amisulpride comparison and the TEAS-by-drug interaction are exploratory, and the trial was not designed to establish superiority, equivalence, or non-inferiority between the two drugs. All participants receive active pharmacological prophylaxis, the study cannot evaluate either drug against no prophylaxis or the combined intervention against neither intervention.

The 48-h primary endpoint was selected to capture clinically meaningful PONV beyond the immediate post-anesthesia care unit period. PONV after gynecologic laparoscopic surgery may persist into the early ward period and affect oral intake, mobilization, patient comfort, and perceived recovery (Zhao et al., 2025). Although the primary outcome captures the overall occurrence of PONV, the prespecified secondary outcomes will provide complementary information on symptom burden, treatment requirements, and functional recovery (Wengritzky, Mettho, Myles, Burke and Kakos, 2010). Assessments of nausea severity, PONV severity, rescue antiemetic use, postoperative pain, QoR-15 scores, patient satisfaction, recovery milestones, and length of hospital stay will help determine whether any observed treatment effect extends beyond event reduction to improvements in patient-centered recovery.

Several design features are incorporated to strengthen the internal validity of the trial. Randomization will be stratified by study center, and outcome assessment and statistical analysis will be performed under blinded conditions. Because the TEAS operator must adjust stimulation intensity according to participant tolerance, operator blinding is not feasible; however, the operator will have no role in postoperative follow-up, outcome assessment, data management, or statistical analysis. An identical-appearing sham device with no electrical output will be used. Participants will receive a standardized, neutral explanation of possible stimulation sensations, and the credibility of participant blinding will be formally assessed at the end of the 48-h follow-up. Perioperative anesthetic and analgesic management will be standardized as much as possible. Relevant perioperative variables, including the use of neuromuscular blockade reversal agents, will be recorded given their potential influence on the risk of PONV (Tramer and Fuchs-Buder, 1999).

## Limitations

This trial has several limitations. First, the TEAS stimulation protocol, including stimulation site, treatment duration, timing, and intensity adjustment, is based on prior literature and clinical feasibility, which may not represent the optimal regimen for all patients. Second, although the study population is clinically homogeneous, the inclusion of only women undergoing gynecologic laparoscopic surgery under general anesthesia may limit the generalizability of the findings to other surgical populations, anesthesia techniques, or patients with different baseline risks of PONV. Third, although the 2 × 2 factorial design permits estimation of both comparisons and assessment of interaction, the sample size calculation was powered for the TEAS-versus-sham comparison. The dexamethasone-versus-amisulpride comparison and the TEAS-by-drug interaction may therefore be underpowered and will be interpreted as exploratory. Fourth, sustained sensation during active TEAS may allow participants to infer their allocation, potentially influencing self-reported outcomes such as nausea severity, patient satisfaction, and QoR-15 scores through treatment expectations.

In summary, this multicenter, randomized, sham-controlled 2 × 2 factorial trial builds on previous randomized evidence supporting TEAS for PONV prevention in gynecologic laparoscopic surgery and extends this evidence by evaluating TEAS across two pharmacologically distinct prophylactic regimens. The study is designed primarily to evaluate the TEAS-versus-sham comparison and to generate prespecified exploratory evidence regarding the dexamethasone-versus-amisulpride comparison and the TEAS-by-drug interaction. The findings may inform integrated, multimodal strategies for PONV prevention in women undergoing gynecologic laparoscopic surgery.
